# Supplementary material for: Omega-3 Fatty Acid Intake during Pregnancy and Child Neuropsychological Development: A Multi-Centre Population-Based Birth Cohort Study in Spain
Source: Nutrients. 2022 Jan 25;14(3):518. doi: 10.3390/nu14030518 (PMC8838693; doi:10.3390/nu14030518)
Supplement: Supplementary file 1 [file nutrients-14-00518-s001.zip › nutrients-1537393-supplementary.pdf]

# Supplementary Material

**Supplementary Figure S1.** Directed Acyclic Graph (DAG) for investigating causal pathways between Child Neuropsychological Outcome and Maternal Dietary omega-3 intake during pregnancy.

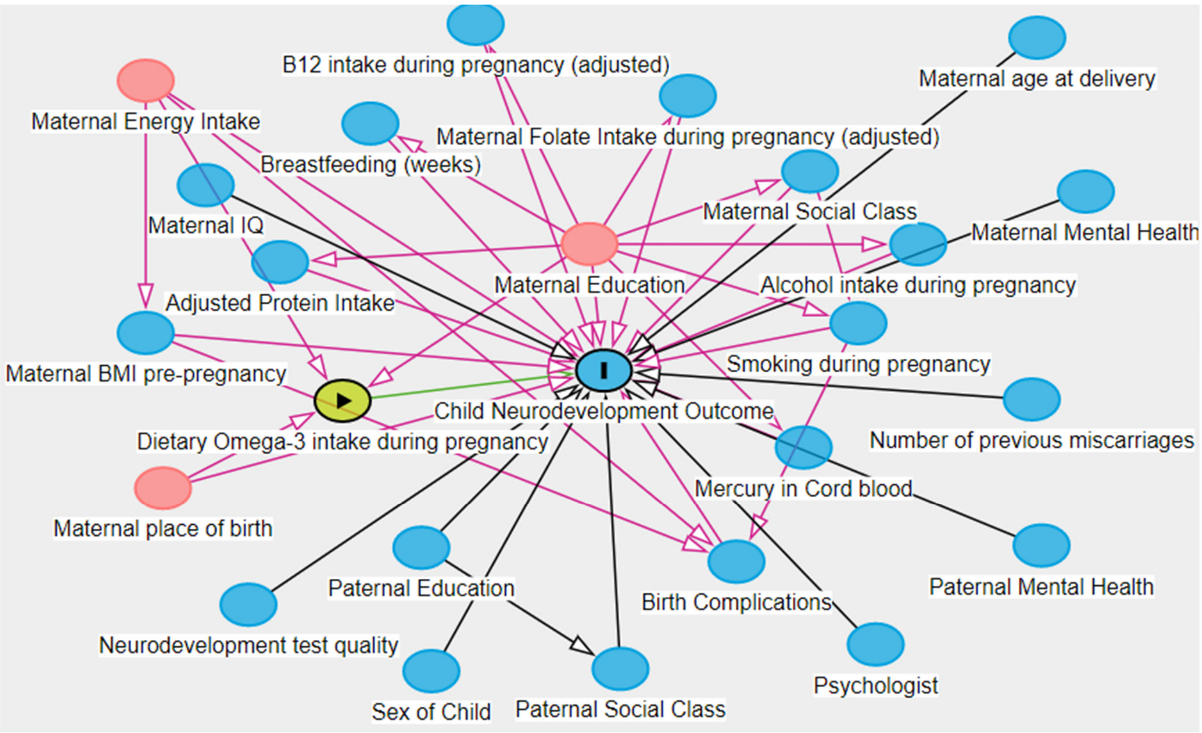

**Supplementary Table S1.** Extended Baseline Characteristics of the study participants according to the quartiles of dietary omega-3 fatty acids Consumption in the First Trimester of Pregnancy.

| | Quartiles of Dietary Omega[b] 3 Intake at 1st Trimester[*] | | | | p values |
|---|---|---|---|---|---|
| | 1 | 2 | 3 | 4 | |
| | (n=645)[a] | (n=645)[a] | (n=645)[a] | (n=647)[a] | |
| Father Education Level, n (%) | | | | 0.535 | |
| Primary or Less | 240 (37.68) | 217 (33.85) | 235 (36.60) | 226 (35.15) | |
| Secondary School | 271 (42.54) | 275 (42.90) | 277 (43.15) | 292 (45.41) | |
| University or More | 126 (19.78) | 149 (23.24) | 130 (20.25) | 125 (19.44) | |
| Social Class of Father, n (%) | | | | | 0.106 |
| Highly Skilled | 120 (19.17) | 145 (22.83) | 121 (18.94) | 111 (17.82) | |
| Non-Manual | 92 (14.70) | 104 (16.38) | 118 (18.47) | 119 (19.10) | |
| Manual | 414 (66.13) | 386 (60.79) | 400 (62.60) | 393 (63.08) | |
| Living situation , n (%) | | | | | 0.821 |
| Live with father's child | 630 (97.67) | 632 (97.98) | 636 (98.15) | 641 (99.07) | |
| Live with partner. | 1 (0.16) | 1 (0.15) | 1 (0.15) | 0 (0) | |
| Live with her parents | 11 (1.71) | 9 (1.40) | 7 (1.08) | 5 (0.77) | |
| Live Alone | 1 (016) | 2 (0.31) | 3 (0.46) | 0 (0) | |
| Others | 2 (0.31) | 1 (0.16) | 1 (0.15) | 1 (0.15) | |
| Mental Health of Mother[c], n (%) | | | | | 0.657 |
| <42 | 82 (25.79) | 73 (21.41) | 98 (26.42) | 90 (26.01) | |
| 42-48 | 80 (25.16) | 102 (29.91) | 85 (22.91) | 82 (23.70) | |
| 48-56 | 76 (23.90) | 84 (24.63) | 92 (24.80) | 83 (23.99) | |
| >56 | 80 (25.16) | 82 (24.05) | 96 (25.88) | 91 (26.30) | |
| Mental Health of Father[c], n (%) | | | | | 0.355 |
| <42 | 92 (24.47) | 76 (19.14) | 101 (23.60) | 112 (25.87) | |
| 42-48 | 89 (23.67) | 112 (28.21) | 119 (27.80) | 121 (27.94 | |
| 48-55 | 88 (23.40) | 96 (24.18) | 103 (24.07) | 99 (22.86) | |
| >55 | 107 (28.46) | 113 (28.46) | 105 (24.53) | 101 (23.33) | |
| Maternal IQ , mean (SD) | 9.81 (3.02) | 9.86 (2.98) | 10.13 (3.08) | 9.95 (2.79) | 0.417 |
| Paternal IQ , mean (SD) | 9.56 (2.96) | 9.75 (3.09) | 9.97 (3.01) | 10.02 (2.75) | 0.721 |
| Paternal smoking in presence of child[d], n (%) | | | | | 0.417 |
| Yes | 29 (14.15) | 34 (17.26) | 26 (11.93) | 27 (12.68) | |
| No | 176 (85.85) | 163 (82.74) | 192 (88.07) | 186 (87.32) | |
| Protein Intake (g/day)[b], mean (SD) | 89.33 (12.65) | 93.14 (11.88) | 96.48 (11.67) | 102.24 (14.57) | 0.001 |
| B12 intake (mg/day)[b] , mean (SD) | 18.53 (167.37) | 15.00 (63.80) | 10.75 (18.18) | 18.14 (74.81) | 0.448 |
| Iron intake (mg/day)[b] , mean (SD) | 16.48 (12.97) | 16.56 (12.44) | 15.97 (12.17) | 16.18 (10.33) | 0.802 |
| Folate intake (mg/day)[b] , mean (SD) | 1953.46 (2647.17) | 1935.31 (2644.05) | 2116.75 (2718.40) | 2181.06 (2842.70) | 0.277 |
| Omega-6 intake (g/day)[b] , mean (SD) | 11.75 (3.47) | 12.30 (3.01) | 12.79 (3.01) | 13.39 (3.33) | 0.001 |
| Dietary Omega-6 /-3 ratio[b], mean (SD) | 10.62 (3.33) | 9.11 (2.20) | 8.31 (1.92) | 6.96 (1.86) | 0.001 |
| DHA intake (g/day)[b], mean (SD) | 0.12 (0.06) | 0.19 (0.07) | 0.26 (0.09) | 0.43 (0.21) | 0.001 |
| Omega-3 supplementation during pregnancy, n (%) | | | | | 0.349 |
| No | 619 (95.97) | 609 (94.42) | 618 (95.37) | 608 (93.97) | |
| Yes | 26 (4.03) | 36 (5.58) | 30 (4.63) | 39 (6.03) | |
| Dietary + Supplementation Omega 3 intake mean (SD) (g/day)[b] | 3.399 (17.98) | 4.58 (16.91) | 4.44 (19.29) | 5.44 (19.02) | 0.258 |
| aMED Score , mean (SD) | 3.48 (1.45) | 3.81 (1.57) | 4.08 (1.67) | 4.22 (1.50) | 0.001 |

| | | | | | |
|---|---|---|---|---|---|
| Preterm | | | | | 0.836 |
| Yes | 32 (5.24) | 26 (4.25) | 27 (4.33) | 29 (4.73) | |
| No | 579 (94.76) | 586 (95.75) | 597 (95.67) | 584 (95.27) | |
| Country of birth of mother, n (%) | | | | | 0.001 |
| Spain | 550 (85.27) | 588 (91.30) | 614 (94.75) | 612 (94.88) | |
| Other | 95 (14.73) | 56 (8.70) | 34 (5.25) | 33 (5.12) | |
| Cohort Location, n (%) | | | | | 0.001 |
| Asturias | 103 (15.97) | 95 (14.73) | 115 (17.75) | 169 (26.12) | |
| Gipuzkoa | 183 (28.37) | 170 (26.36) | 155 (23.92) | 119 (18.39) | |
| Sabadell | 123 (19.07) | 149 (23.10) | 187 (28.86) | 195 (30.14) | |
| Valencia | 236 (36.59) | 231 (35.81) | 191 (29.48) | 164 (25.35) | |
| Mercury in Cord blood (μg/L), mean (SD) | 8.86 (6.81) | 11.06 (9.42) | 11.62 (9.77) | 12.31 (9.12) | 0.001 |
| Omega 3 % in cord blood | | | | | |
| ALA in %, mean (SD) | 0.09 (0.07) | 0.11 (0.11) | 0.11 (0.12) | 0.10 (0.09) | 0.256 |
| EPA in %, mean (SD) | 0.23 (0.19) | 0.23 (0.17) | 0.24 (0.20) | 0.23 (0.15) | 0.993 |
| DHA in %, mean (SD) | 5.10 (1.72) | 5.15 (1.65) | 5.05 (1.59) | 5.14 (1.70) | 0.915 |
| ***Child Characteristics*** | | | | | |
| Omega 3 intake at 4 year of age , mean (SD) | 0.96 (0.21) | 1.01 (0.21) | 1.03 (0.20) | 1.05 (0.21) | 0.000 |
| Low birthweight, n (%) | | | | | |
| 0.347 | | | | | |
| Yes | 582 (95.10) | 585 (95.12) | 584 (93.59) | 590 (95.78) | |
| No | 30 (4.90) | 30 (4.88) | 40 (6.41) | 26 (4.22) | |

[*]**1st quartile:** *<1.262g/day*. **2nd quartile:** *1.262g-1.440g/day*. **3rd quartile:** *1.440g-1.657g/day*. **4th quartile:** *>1.657g/day*
[a] Some categories may be missing values so therefore totals may not match total number of subjects
[b]Adjusted for energy intake
[c]Global Severity Index the Symptom Checklist-90-R, T Score
[d]Yes = "often"+"sometimes". No = "no"+hardley ever"

**Supplementary Table S2 .** Baseline Characteristics of the study participants according to the quartiles of dietary omega-3 fatty acids Consumption in the 3rd Trimester of Pregnancy.

| | Quartiles of Dietary Omega-3[b] Intake at 3rd Trimester[*] | | | | p values |
|---|---|---|---|---|---|
| | 1<br>(n=612)[a] | 2<br>(n=610)[a] | 3<br>(n=611)[a] | 4<br>(n=610)[a] | |
| Age (years)[c], mean (SD) | 31.36 (4.53) | 31.89 (4.16) | 32.19 (4.19) | 32.35 (4.00) | 0.001 |
| Mother Education Level, n (%) | | | | | 0.001 |
| Primary or Less | 186 (30.44) | 134 (22.00) | 147 (24.14) | 125 (20.49) | |
| Secondary School | 237 (38.79) | 246 (40.39) | 241 (29.57) | 289 (47.38) | |
| University or More | 188 (30.77) | 229 (37.60) | 221 (36.29) | 196 (32.13) | |
| Father Education Level, n (%) | | | | | 0.005 |
| Primary or Less | 252 (41.65) | 192 (31.79) | 209 (34.60) | 203 (33.28) | |
| Secondary School | 228 (37.69) | 281 (46.52) | 279 (46.19) | 284 (46.56) | |
| University or More | 125 (20.66) | 131 (21.69) | 116 (19.21) | 123 (20.16) | |
| Social Class of Mother, n (%) | | | | 0.000 | |
| Highly Skilled | 112 (18.33) | 147 (24.10) | 156 (25.53) | 109 (17.87) | |
| Non-Manual | 145 (23.73) | 160 (26.23) | 145 (23.73) | 187 (20.66) | |
| Manual | 354 (57.94) | 303 (49.67) | 310 (50.74) | 314 (51.48) | |
| Social Class of Father, n (%) | | | | | 0.215 |
| Highly Skilled | 106 (17.79) | 134 (22.60) | 119 (19.73) | 114 (19.32) | |
| Non-Manual | 99 (16.61) | 95 (16.02) | 101 (16.75) | 118 (20.00) | |
| Manual | 391 (65.90) | 364 (61.38) | 383 (63.52) | 358 (60.68) | |
| Living situation , n (%) | | | | | 0.288 |
| Live with father's child | 598 (97.71) | 599 (98.20) | 598 (98.03) | 604 (99.02) | |
| Live with partner | 1 (0.16) | 1 (0.16) | 1 (0.16) | 0 (0.00) | |
| Live with her parents | 9 (1.47) | 9 (1.48) | 5 (0.82) | 6 (0.98) | |
| Live Alone | 1 (0.16) | 1 (0.16) | 4 (0.66) | 0 (0.00) | |
| Others | 3 (0.49) | 0 (0.00) | 2 (0.33) | 0 (0.00) | |
| Mental Health of Mother[d], n (%) | | | | | 0.970 |
| <42 | 82 (25.62) | 87 (25.44) | 77 (22.71) | 95 (26.32) | |
| 42-48 | 87 (27.19) | 83 (24.27) | 86 (25.37) | 87 (24.10) | |
| 48-56 | 75 (23.44) | 83 (24.27) | 83 (24.48) | 88 (24.38) | |
| >56 | 76 (23.75) | 89 (26.02) | 93 (27.43) | 91 (25.21) | |
| Mental Health of Father[d], n (%) | | | | | 0.782 |
| <42 | 84 (22.22) | 93 (23.85) | 94 (23.15) | 103 (23.57) | |
| 42-48 | 104 (27.51) | 103 (26.41) | 102 (25.12) | 125 (28.60) | |
| 48-55 | 82 (21.69) | 92 (23.59) | 98 (24.14) | 110 (25.17) | |
| >55 | 108 (28.57) | 102 (26.15) | 112 (27.59) | 99 (22.65) | |
| Maternal IQ , mean (SD) | 9.58 (3.04) | 10.24 (3.10) | 10.08 (2.85) | 9.88 (2.84) | 0.009 |
| Paternal IQ , mean (SD) | 9.98 (3.04) | 9.78 (2.60) | 9.67 (3.26) | 9.87 (2.84) | 0.907 |
| Smoking during pregnancy, n (%) | | | | | 0.247 |
| Yes | 115 (18.82) | 98 (16.12) | 91 (14.92) | 110 (18.06) | |
| No | 496 (81.18) | 510 (83.88) | 519 (85.08) | 499 (81.94) | |
| Paternal smoking in presence of child[e], n (%) | | | | | 0.247 |
| Yes | 26 (13.20) | 34 (16.83) | 35 (15.70) | 21 (10.40) | |
| No | 171 (86.80) | 168 (83.17) | 188 (84.30) | 181 (89.60) | |
| Alcohol Consumption during Third Trimester, n (%) | | | | | 0.009 |
| Yes | 415 (67.81) | 369 (60.49) | 369 (60.39) | 363 (59.51) | |
| No | 197 (32.19) | 241 (39.51) | 242 (39.61) | 247 (40.49) | |
| Energy intake (kcals/day), mean (SD) | 2083.84 (568.14) | 2069.14 (522.04) | 2091.94 (550.49) | 2065.92 (557.35) | 0.822 |
| Protein Intake (g/day)[b], mean (SD) | 89.21 (12.23) | 92.07 (11.93) | 95.44 (12.24) | 101.46 (13.99) | 0.000 |
| B12 intake (mg/day)[b], | 16.66 | 15.97 | 18.50 | 20.45 | 0.738 |

| | | | | | |
|---|---|---|---|---|---|
| mean (SD) | (74.76) | (67.51) | (78.09) | (85.18) | |
| Iron intake (mg/day)[b], 0.455 mean (SD) | 32.29 (29.05) | 31.43 (30.01) | 30.00 (28.97) | 32.36 (28.43) | |
| Folate intake (mg/day)[b], mean (SD) | 1813.56 (2890.11) | 1652.19 (2696.95) | 1575.29 (2563.99) | 1954.52 (3041.79) | 0.083 |
| Omega-6 intake (g/day)[b] , mean (SD) | 11.14 (3.44) | 11.82 (3.31) | 11.96 (2.57) | 12.50 (3.02) | 0.001 |
| Dietary Omega-6 /-3 ratio[b] , mean (SD) | 10.39 (3.59) | 9.01 (2.57) | 8.00 (1.71) | 6.74 (1.66) | 0.001 |
| DHA intake (grams)[b], mean (SD) | 0.13 (0.06) | 0.19 (0.07) | 0.26 (0.09) | 0.43 (0.20) | 0.001 |
| Omega-3 supplementation during Pregnancy, n (%) | | | | | 0.312 |
| Yes | 23 (3.76) | 31 (5.08) | 36 (5.89) | 35 (5.74) | |
| No | 589 (96.24) | 579 (94.92) | 575 (94.11) | 575 (94.26) | |
| Dietary + Supplementation Omega 3 intake (g/day), mean (SD) [b] | 13.81 (57.49) | 13.35 (53.17) | 12.36 (46.05) | 13.52 (48.34) | 0.965 |
| aMED Score , mean (SD) | 3.47 (1.45) | 3.75 (1.51) | 3.97 (1.61) | 4.16 (1.51) | 0.001 |
| Preterm, n (%) | | | | | 0.287 |
| Yes | 31 (5.15) | 21 (3.51) | 21 (3.52) | 22 (2.64) | |
| No | 571 (94.85) | 578 (96.49) | 575 (96.48) | 583 (96.36) | |
| Mother BMI pre-pregnancy, mean (SD) | 23.52 (4.57) | 23.35 (4.15) | 23.69 (4.19) | 23.70 (4.36) | 0.450 |
| Number of previous miscarriages , n (%) | | | | | 0.198 |
| 0 | 462 (75.61) | 484 (79.34) | 454 (74.30) | 468 (76.72) | |
| 1 or more | 149 (24.39) | 126 (20.66) | 157 (25.70) | 142 (23.28) | |
| Country of birth of mother, n (%) | | | | | 0.001 |
| Spain | 531 (86.76) | 567 (92.95) | 558 (91.78) | 580 (95.24) | |
| Other | 81 (13.24) | 43 (7.05) | 50 (8.22) | 29 (4.76) | |
| Cohort Location, n (%) | | | | | 0.001 |
| Asturias | 89 (14.54) | 94 (15.41) | 109 (17.84) | 153 (25.08) | |
| Gipuzkoa | 157 (25.65) | 172 (28.20) | 152 (24.88) | 115 (18.85) | |
| Sabadell | 128 (20.92) | 144 (23.61) | 167 (27.33) | 176 (28.85) | |
| Valencia | 238 (38.89) | 200 (32.79) | 183 (29.95) | 166 (27.21) | |
| Mercury in Cord blood (µg/L), mean (SD) | 8.66 (7.50) | 10.28 (8.09) | 11.58 (8.97) | 13.56 (10.30) | 0.001 |
| Omega 3 % in cord blood , mean (SD) | | | | | |
| ALA in cord blood | 0.10 (0.10) | 0.10 (0.10) | 0.11 (0.11) | 0.10 (0.10) | 0.888 |
| EPA in cord blood | 0.22 (0.18) | 0.25 (0.21) | 0.24 (0.18) | 0.23 (0.15) | 0.365 |
| DHA in cord blood | 4.97 (1.64) | 5.25 (1.64) | 5.12 (1.76) | 5.11 (1.58) | 0.372 |
| Cord Blood Omega 6(AA)/ Omega 3 (EPA+DHA) ratio, mean (SD) | 3.04 (0.911) | 2.87 (0.79) | 2.89 (0.79) | 2.88 (0.79) | 0.091 |
| Breastfeeding (weeks), n (%) | | | | | 0.690 |
| 0 | 84 (15.33) | 79 (14.18) | 72 (12.70) | 89 (15.48) | |
| >0-16 | 152 (27.74) | 138 (24.78) | 137 (24.16) | 142 (24.70) | |
| >16-24 | 79 (14.42) | 96 (17.24) | 91 (16.05) | 91 (15.83) | |
| >24 | 233 (42.52) | 244 (43.81) | 267 (47.09) | 253 (44.00) | |
| ***Child Characteristics*** | | | | | |
| Sex , n (%) | | | | | 0.896 |
| Female | 290 (47.93) | 299 (49.34) | 297 (49.34) | 290 (47.62) | |
| Male | 315 (52.07) | 307 (50.66) | 305 (50.66) | 319 (52.38) | |
| Omega 3 intake at 4 year of age, mean (SD) | 0.97 (0.21) | 1.00 (0.19) | 1.02 (0.20) | 1.06 (0.21) | 0.001 |
| Birthweight , n (%) | | | | | 0.401 |
| <3000g | 162 (26.91) | 167 (27.65) | 159 (26.63) | 163 (26.85) | |
| 3000-3500g | 276 (45.85) | 277 (45.86) | 273 (45.73) | 250 (41.19) | |
| <3500g | 164 (27.24) | 160 (26.49) | 165 (27.64) | 194 (31.96) | |
| Low birthweight , n (%) | | | | | 0.873 |

| | | | | |
|---|---|---|---|---|
| Yes | 29 (4.82) | 31 (5.13) | 25 (4.19) | 27 (4.45) |
| No | 573 (95.18) | 573 (94.87) | 572 (95.81) | 580 (95.55) |

[*]**1st quartile:** *<1.219g*. **2nd quartile:** *1.219g-1.399g*. **3rd quartile:** *1.399g-1.599g*. **4th quartile:** *>1.599g*
[a] Some categories may be missing values so therefore totals may not match total number of subject
[b]Adjusted for energy intake
[c]Maternal Age at Delivery
[d]Global Severity Index the Symptom Checklist-90-R, T Score
[e]Yes = "often"+"sometimes". No = "no"+hardley ever"

**Supplementary Table S3.** Mean test scores and standard deviation (SD) from Bayley Scales of Infant Development at 1 Year of Age according to maternal omega-3 intake frequency during the first and third trimester of pregnancy.

| | Quartiles of Dietary Omega-3[b] Intake at 1st Trimester | | | | p values |
|---|---|---|---|---|---|
| | 1 (n=539) | 2 (n=555) | 3 (n=551)[a] | 4 (n=552) | |
| Mental Score[a] | 99.83 (15.12) | 98.90 (15.38) | 100.75 (15.01) | 100.12 (15.31) | 0.235 |
| Motor Score[a] | 100.31 (15.10) | 99.43 (15.34) | 99.78 (14.78) | 99.87 (15.18) | 0.814 |
| | **Quartiles of Dietary Omega-3[b] Intake at 3rd Trimester** | | | | **p values** |
| | 1 (n = 528) | 2 (n = 545) | 3 (n = 549) | 4 (n = 542) | |
| Mental Score[a] | 99.83 (15.63) | 100.04 (15.12) | 99.82 (15.57) | 99.96 (14.50) | 0.994 |
| Motor Score[a] | 100.67 (15.51) | 99.94 (15.30) | 99.92 (14.83) | 98.59 (14.69) | 0.148 |

[a]Index score centered to 100 and standard deviation of 15
[b]Adjusted for energy intake

**Supplementary Table S4.** Extended mean test scores and standard deviation (SD) from McCarthy Scales of Children's Abilities (MSCA) at 4 year of age according to maternal omega-3 intake frequency during the first and third trimester of pregnancy.

| | Quartiles of Dietary Omega-3[b] Intake at 1st Trimester | | | | p values |
|---|---|---|---|---|---|
| | 1 (n=422) | 2 (n=444) | 3 (n=468) | 4 (n=461) | |
| Perceptive Performance[a] | 99.24 (14.75) | 101.12 (15.18) | 100.13 (14.53) | 100.42 (14.98) | 0.311 |
| Numerical[a] | 99.00 (14.52) | 100.69 (15.89) | 100.59 (14.11) | 100.73 (14.98) | 0.253 |
| Memory[a] | 98.67 (14.57) | 100.65 (14.80) | 100.55 (14.28) | 100.74 (15.98) | 0.127 |
| Motor[a] | 99.66 (15.66) | 100.89 (15.30) | 100.13 (14.16) | 100.08 (14.59) | 0.674 |
| | **Quartiles of Dietary Omega-3[b] Intake at 3rd Trimester** | | | | **p values** |
| | 1 (n=419) | 2 (n=426) | 3 (n=448) | 4 (n=467) | |
| General Cognitive[a] | 98.78 (15.68) | 100.47 (14.19) | 100.98 (14.63) | 100.62 (14.59) | 0.132 |
| Verbal[a] | 99.50 (15.11) | 100.29 (14.49) | 100.98 (15.00) | 99.80 (15.06) | 0.476 |
| Perceptive Performance[a] | 98.67 (15.95) | 100.49 (14.04) | 100.69 (14.68) | 100.96 (14.48) | 0.096 |
| Numerical[a] | 98.71 (15.31) | 100.51 (14.72) | 100.62 (14.94) | 100.90 (14.51) | 0.123 |
| Memory[a] | 99.22 (14.66) | 100.50 (15.14) | 100.23 (15.14) | 100.44 (14.92) | 0.565 |
| Motor[a] | 98.83 (15.44) | 100.52 (13.75) | 100.64 (14.82) | 100.64 (15.32) | 0.209 |
| Executive Function[a] | 98.77 (15.73) | 100.50 (13.92) | 101.09 (14.96) | 100.54 (14.62) | 0.115 |

[a]Score centered to 100 and standard deviation of 15

[b]Adjusted for energy intake

**Supplementary Table S5.** Extended mean test scores and standard deviation (SD) from Attention Network Test of Children's Abilities 7 year of age according to maternal omega-3 intake frequency during the first and third trimester of pregnancy.

| | Quartiles of Dietary Omega-3[b] Intake at 1st Trimester | | | | p values |
|---|---|---|---|---|---|
| | 1 (n=379) | 2 (n=411) | 3 (n=444) | 4 (n=421) | |
| Alerting Network ANT | 48.85 (103.48) | 52.46 (98.12) | 48.76 (92.34) | 49.71 (86.21) | 0.934 |
| Alerting Johnson | 52.41 (128.34) | 56.00 (138.79) | 50.58 (116.00) | 54 (115.17) | 0.922 |
| Orienting network ANT | 32.02 (89.62) | 34.77 (83.84) | 32.19 (86.81) | 42.95 (87.88) | 0.225 |
| Orienting Johnson | 27.39 (117.29) | 32.84 (109.30) | 26.43 (117.48) | 41.16 (113.96) | 0.224 |
| Conflict network ANT | 84.84 (81.21) | 73.03 (77.82) | 82.69 (85.31) | 79.94 (80.01) | 0.183 |
| | Quartiles of Dietary Omega-3[b] Intake at 3rd Trimester | | | | p values |
| | 1 (n=372) | 2 (n=401) | 3 (n=424) | 4 (n=426) | |
| Alerting network ANT | 48.11 | 51.58 | 49.21 | 50.88 | 0.955 |
| Alerting Johnson | 56.42 (132.55) | 48.90 (124.59) | 50.22 (123.35) | 58.03 (120.30) | 0.661 |
| Orienting network ANT | 33.57 (85.47) | 34.27 (81.71) | 36.09 (87.56) | 38.10 (94.23) | 0.884 |
| Orienting Johnson | 24.96 (115.41) | 33.22 (110.93) | 30.70 (115.27) | 38.25 (117.93) | 0.432 |
| HRT-SE | 308.69 (85.32) | 308.89 (80.85) | 309.01 (79.83) | 301.32 (83.44) | 0.450 |
| Conflict network ANT | 78.94 (81.88) | 78.92 (80.92) | 79.58 (74.73) | 84.61 (87.83) | 0.701 |

[b]Adjusted for energy intake

**Supplementary Table S6.** Extended multivariable regression analysis: Associations between maternal omega-3 consumption in the first trimester of pregnancy and child's scores on Bayley Scales of Infant Development (BSID) at 1 year of age, McCarthy Scales of Children's Abilities (MSCA) at 4 year of age and Attention Network Test of Children's Abilities (ANT) at 7 year of age.

| Neuropsychological Outcome | | No. of Subjects | Difference in child's neuropsychological score | | | | | |
|---|---|---|---|---|---|---|---|---|
| | | | Minimally adjusted* | | | Fully adjusted* | | |
| | | | β | (95% CI) | P | β | (95% CI) | P |
| **BSID** | | | | | | | | |
| Mental Score | | | | | | | | |
| | First quartile | 520 | ref | | | ref | | |
| | Second quartile | 530 | -1.16 | (-2.72, 0.40) | 0.145 | -1.00 | (-2.60, 0.60) | 0.220 |
| | Third quartile | 523 | 0.51 | (-1.06, 2.08) | 0.524 | 0.45 | (-1.17, 2.07) | 0.586 |
| | Fourth quartile | 531 | 0.37 | (-1.20, 1.95) | 0.640 | 0.33 | (-1.29, 1.98) | 0.679 |
| Motor Score | | | | | | | | |
| | First quartile | 520 | ref | | | ref | | |
| | Second quartile | 530 | -1.01 | (-2.72, 0.70) | 0.247 | -0.66 | (-2.40, 1.08) | 0.459 |
| | Third quartile | 523 | -0.77 | (-2.49, 0.96) | 0.383 | -0.61 | (-2.38, 1.16) | 0.500 |
| | Fourth quartile | 531 | -0.47 | (-2.20, 1.26) | 0.597 | -0.25 | (-2.02, 1.53) | 0.785 |
| **MSCA** | | | | | | | | |
| Perceptive Performance | | | | | | | | |
| | First quartile | 409 | ref | | | ref | | |
| | Second quartile | 425 | 1.79 | (-0.08, 3.66) | 0.061 | 1.35 | (-0.49, 3.21) | 0.152 |
| | Third quartile | 443 | 0.86 | (-1.00, 2.71) | 0.366 | 0.39 | (-1.46, 2.24) | 0.678 |
| | Fourth quartile | 436 | 1.33 | (-0.55, 3.21) | 0.164 | 0.94 | (-0.95, 2.82) | 0.330 |
| Numerical | | | | | | | | |
| | First quartile | 409 | ref | | | ref | | |
| | Second quartile | 425 | 1.59 | (-0.35, 3.53) | 0.108 | 1.02 | (-0.90, 2.95) | 0.298 |
| | Third quartile | 443 | 1.62 | (-0.31, 3.54) | 0.100 | 0.95 | (-0.97, 2.88) | 0.331 |
| | Fourth quartile | 436 | 1.75 | (-0.19, 3.70) | 0.078 | 1.00 | (-0.95, 2.96) | 0.313 |
| Memory | | | | | | | | |
| | First quartile | 409 | ref | | | ref | | |
| | Second quartile | 425 | 1.89 | (-0.06, 3.83) | 0.057 | 1.4 | (-0.55, 3.35) | 0.160 |
| | Third quartile | 443 | 2.00 | (0.08, 3.93) | 0.042 | 1.63 | (-0.32, 3.58) | 0.101 |
| | Fourth quartile | 436 | 2.15 | (0.19, 4.09) | 0.031 | 1.89 | (-0.09, 3.87) | 0.061 |
| Motor | | | | | | | | |
| | First quartile | 409 | ref | | | ref | | |
| | Second quartile | 425 | 1.02 | (-0.90, 2.94) | 0.296 | 0.78 | (-1.17, 2.72) | 0.432 |
| | Third quartile | 443 | 0.36 | (-1.54, 2.26) | 0.713 | 0.01 | (-1.93, 1.96) | 0.988 |
| | Fourth quartile | 436 | 0.49 | (-1.44, 2.40) | 0.625 | 0.002 | (-1.97, 1.98) | 0.998 |
| **ANT** | | | | | | | | |
| Alerting network | | | | | | | | |
| | First quartile | 363 | ref | | | ref | | |
| | Second quartile | 392 | 3.92 | (-9.36, 17.21) | 0.563 | 6.59 | (-7.21, 20.40) | 0.349 |
| | Third quartile | 421 | 0.45 | (-12.65, 13.55) | 0.947 | 1.83 | (-11.83, 15.50) | 0.792 |
| | Fourth quartile | 398 | 1.72 | (-11.66, 15.10) | 0.801 | 2.90 | (-11.17, 16.97) | 0.686 |
| Alerting Johnson | | | | | | | | |
| | First quartile | 363 | ref | | | ref | | |
| | Second quartile | 392 | 3.83 | (-13.62, 21.27) | 0.667 | 4.28 | (-13.84, 22.39) | 0.644 |
| | Third quartile | 421 | -1.90 | (-19.11, 15.30) | 0.828 | -0.44 | (-18.38, 17.49) | 0.961 |
| | Fourth quartile | 398 | 1.56 | (-16.01, 19.13) | 0.862 | 1.58 | (-16.89, 20.05) | 0.867 |
| Orienting network ANT | | | | | | | | |
| | First quartile | 363 | ref | | | ref | | |
| | Second quartile | 392 | 2.98 | (-9.19, 15.15) | 0.631 | 4.52 | (-8.11, 17.14) | 0.483 |
| | Third quartile | 421 | 1.08 | (-10.92, 13.09) | 0.860 | 1.45 | (-11.04, 13.95) | 0.820 |
| | Fourth quartile | 398 | 12.06 | (-0.20, 24.32) | 0.054 | 12.33 | (-0.54, 25.20) | 0.061 |
| Orienting Johnson | | | 0.687 | | | | | 0.766 |
| | First quartile | 363 | ref | | | ref | | |
| | Second quartile | 392 | 5.60 | (-10.44, 21.64) | 0.493 | 5.63 | (-10.94, 22.19) | 0.505 |
| | Third quartile | 421 | -0.59 | (-16.41, 15.24) | 0.942 | -3.57 | (-19.96, 12.83) | 0.670 |
| | Fourth quartile | 398 | 13.58 | (-2.58, 29.74) | 0.099 | 14.36 | (-2.52, 31.25) | 0.095 |
| Conflict network ANT | | | | | | | | |
| | First quartile | 363 | ref | | | ref | | |
| | Second quartile | 392 | -12.26 | (-23.52, -1.01) | 0.033 | -13.83 | (-25.48, -2.18) | 0.020 |
| | Third quartile | 421 | -3.50 | (-14.60, 7.60) | 0.536 | -3.19 | (-14.72, 8.34) | 0.587 |
| | Fourth quartile | 398 | -4.55 | (-15.88, 6.79 | 0.432 | -4.09 | (-15.97, 7.78) | 0.499 |

[a]Multivariate linear regression models adjusted for sex of child, child's age at testing, cohort, quality of the test (none for ANT), and maternal energy intake.
[b] Multivariate linear regression models adjusted additionally for birthweight of the child, gestational age, duration of breastfeeding, maternal alcohol consumption, maternal education, maternal smoking, maternal social class, pre-pregnancy BMI, number of pregnancies, number of miscarriages, maternal country of origin.

**Supplementary Table S7.** Multivariable regression analysis: Associations between maternal omega-3 consumption in the third trimester of pregnancy and child's scores on Bayley Scales of Infant Development (BSID) at 1 year of age, McCarthy Scales of Children's Abilities (MSCA) at 4 year of age and Attention Network Test of Children's Abilities (ANT) at 7 year of age.

| Neuropsychological Outcome | | No. of Subjects | Difference in child's neuropsychological score | | | | | |
|---|---|---|---|---|---|---|---|---|
| | | | Minimally adjusted* | | | Fully adjusted* | | |
| | | | β | (95% CI) | P | β | (95% CI) | P |
| **BSID** | | 2104 | | | | | | |
| Mental Score | | | | | *0.000 | | | *0.000 |
| | First quartile | 511 | ref | | | ref | | |
| | Second quartile | 530 | -0.38 | (-1.96, 1.21) | 0.641 | -0.65 | (-2.27, 0.96) | 0.430 |
| | Third quartile | 531 | 0.25 | (-1.34, 1.83) | 0.760 | 0.20 | (-1.41, 1.82) | 0.804 |
| | Fourth quartile | 533 | 0.60 | (-1.00, 2.19) | 0.463 | 0.52 | (-1.12, 2.15) | 0.535 |
| Motor Score | | | | | | | | |
| | First quartile | 511 | ref | | | ref | | |
| | Second quartile | 530 | -1.10 | (-2.83, 0.63) | 0.211 | -0.91 | (-2.66, 0.85) | 0.311 |
| | Third quartile | 531 | -0.66 | (-2.39, 1.07) | 0.454 | -0.80 | (-2.56, 0.95) | 0.369 |
| | Fourth quartile | 533 | -1.84 | (-3.58, -0.10) | 0.039 | -1.56 | (-3.34, 0.21) | 0.084 |
| **MSCA Scores** | | | | | | | | |
| General Cognitive | | | | | | | | |
| | First quartile | 405 | ref | | | ref | | |
| | Second quartile | 417 | 1.57 | (-0.34, 3.48) | 0.107 | 0.40 | (-1.46, 2.25) | 0.67 |
| | Third quartile | 432 | 2.07 | (0.18, 3.96) | 0.032 | 1.84 | (0.009, 3.67) | 0.049 |
| | Fourth quartile | 456 | 2.02 | (0.14, 3.89) | 0.035 | 0.99 | (-0.84, 2.82) | 0.290 |
| Verbal | | | | | | | | |
| | First quartile | 405 | ref | | | ref | | |
| | Second quartile | 417 | 0.73 | (-1.25, 2.70) | 0.470 | -0.22 | (-2.17, 1.73) | 0.823 |
| | Third quartile | 432 | 1.38 | (-0.57, 3.34) | 0.165 | 1.39 | (-0.54, 3.31) | 0.158 |
| | Fourth quartile | 456 | 0.43 | (-1.51, 2.37) | 0.666 | -0.37 | (-2.30, 1.55) | 0.705 |
| Perceptive Performance | | | | | | | | |
| | First quartile | 405 | ref | | | ref | | |
| | Second quartile | 417 | 1.65 | (-0.25, 3.55) | 0.088 | 0.75 | (-1.13, 2.63) | 0.436 |
| | Third quartile | 432 | 1.89 | (0.01, 3.77) | 0.048 | 1.53 | (-0.33, 3.38) | 0.107 |
| | Fourth quartile | 456 | 2.43 | (0.57, 4.30) | 0.001 | 1.56 | (-0.30, 3.42) | 0.099 |
| Numerical | | | | | | | | |
| | First quartile | 405 | ref | | | ref | | |
| | Second quartile | 417 | 1.76 | (-0.21, 3.72) | 0.080 | 0.58 | (-1.37, 2.54) | 0.558 |
| | Third quartile | 432 | 1.82 | (-0.12, 3.77) | 0.066 | 1.51 | (-0.42, 3.44) | 0.126 |
| | Fourth quartile | 456 | 2.36 | (0.43, 4.30) | 0.017 | 1.40 | (-0.53, 3.33) | 0.154 |
| Memory | | | | | | | | |
| | First quartile | 405 | ref | | | ref | | |
| | Second quartile | 417 | 1.24 | (-0.74, 3.22) | 0.218 | 0.28 | (-1.70, 2.26) | 0.785 |
| | Third quartile | 432 | 0.95 | (-1.00, 2.91) | 0.338 | 0.68 | (-1.30, 2.63) | 0.498 |
| | Fourth quartile | 456 | 1.39 | (-0.55, 3.33) | 0.160 | 0.69 | (-1.26, 2.65) | 0.486 |
| Motor | | | | | | | | |
| | First quartile | 405 | ref | | | ref | | |
| | Second quartile | 417 | 1.67 | (-0.27, 3.61) | 0.091 | 1.04 | (-0.93, 3.01) | 0.300 |
| | Third quartile | 432 | 1.69 | (-0.22, 3.61) | 0.083 | 1.28 | (-0.66, 3.22) | 0.196 |
| | Fourth quartile | 456 | 2.05 | (0.15, 3.95) | 0.035 | 1.41 | (-0.53, 3.36) | 0.153 |
| Executive Function | | | | | | | | |
| | First quartile | 405 | ref | | | ref | | |
| | Second quartile | 417 | 1.66 | (-0.28, 3.61) | 0.094 | 0.34 | (-1.55, 2.23) | 0.724 |
| | Third quartile | 432 | 2.18 | (0.26, 4.11) | 0.026 | 1.94 | (0.08, 3.81) | 0.041 |
| | Fourth quartile | 456 | 1.96 | (0.05, 3.87) | 0.044 | 0.66 | (-1.20, 2.53) | 0.485 |
| **ANT** | | | | | | | | |
| Alerting network | | | | | | | | |
| | First quartile | 356 | ref | | | ref | | |
| | Second quartile | 392 | 4.21 | (-9.25, 17.69) | 0.540 | 2.19 | (-11.74, 16.12) | 0.75 |
| | Third quartile | 408 | 2.35 | (-10.96, 15.67) | 0.729 | 1.93 | (-11.84, 15.70) | 0.783 |
| | Fourth quartile | 418 | 3.61 | (-9.72, 16.93) | 0.596 | 2.03 | (-11.77, 15.82) | 0.773 |
| Alerting Johnson | | | | | | | | |
| | First quartile | 356 | ref | | | ref | | |
| | Second quartile | 392 | -6.67 | (-24.38, 11.03) | 0.460 | -11.05 | (-29.35, 7.25) | 0.237 |
| | Third quartile | 408 | -5.26 | (-22.76, 12.24) | 0.556 | -7.44 | (-25.52, 10.64) | 0.420 |

| | | | | | | | | |
|---|---|---|---|---|---|---|---|---|
| | Fourth quartile | 418 | 1.52 | (-15.99, 19.04) | 0.864 | -0.06 | (-18.17, 18.06) | 0.995 |
| Orienting network ANT | | | | | | | | |
| | First quartile | 356 | ref | | | ref | | |
| | Second quartile | 392 | 1.20 | (-11.19, 13.58) | 0.850 | 1.32 | (11.46, 14.10) | 0.839 |
| | Third quartile | 408 | 2.87 | (-9.37, 15.10) | 0.646 | 3.60 | (-9.02, 16.23) | 0.576 |
| | Fourth quartile | 418 | 4.76 | (-7.48, 17.01) | 0.446 | 6.26 | (-6.34, 18.91) | 0.332 |
| Orienting Johnson | | | | | | | | |
| | First quartile | 356 | ref | | | ref | | |
| | Second quartile | 392 | 8.50 | (-7.78, 24.77) | 0.306 | 5.51 | (-11.21, 22.24) | 0.518 |
| | Third quartile | 408 | 5.60 | (-10.48, 21.69) | 0.494 | 4.91 | (-11.61, 21.44) | 0.560 |
| | Fourth quartile | 418 | 13.11 | (-2.98, 29.21) | 0.110 | 13.05 | (-3.50, 29.61) | 0.122 |
| HRT-SE | | | | | | | | |
| | First quartile | 356 | ref | | | ref | | |
| | Second quartile | 392 | -0.85 | (-11.92, 10.22) | 0.880 | 1.19 | (-10.13, 12.50) | 0.837 |
| | Third quartile | 408 | 2.37 | (-8.56, 13.31) | 0.670 | 3.55 | (-7.63, 14.73) | 0.533 |
| | Fourth quartile | 418 | -5.04 | (-15.98, 5.91) | 0.367 | -3.77 | (-14.97, 7.44) | 0.510 |
| Conflict network ANT | | | | | | | | |
| | First quartile | 356 | ref | | | ref | | |
| | Second quartile | 392 | -0.51 | (-11.93, 10.92) | 0.930 | 2.37 | (-9.42, 14.16) | 0.693 |
| | Third quartile | 408 | 0.83 | (-10.46, 12.12) | 0.886 | 1.36 | (-10.29, 13.01) | 0.819 |
| | Fourth quartile | 418 | 5.94 | (-5.36, 17.24) | 0.303 | 7.41 | (-4.27, 19.08) | 0.214 |

[a]Multivariate linear regression models adjusted for sex of child, child's age at testing, cohort, quality of the test (none for ANT), and maternal energy intake.

[b] Multivariate linear regression models adjusted additionally for birthweight of the child, gestational age, duration of breastfeeding, maternal alcohol consumption, maternal education, maternal smoking, maternal social class, pre-pregnancy BMI, number of pregnancies, number of miscarriages, maternal country of origin.
